# Genetic features and phylogenetic relationship analyses of Guizhou Han population residing in Southwest China via 38 X-InDels

**DOI:** 10.7717/peerj.14964

**Published:** 2023-03-08

**Authors:** Yuhang Feng, Ting Wang, Yunteng Yang, Jiangtao You, Kun He, Hongling Zhang, Qiyan Wang, Meiqing Yang, Jiang Huang, Zheng Ren, Xiaoye Jin

**Affiliations:** 1 Shanghai Key Lab of Forensic Medicine, Key Lab of Forensic Science, Ministry of Justice, China, Academy of Forensic Science, Shanghai, China; 2 Department of Forensic Medicine, Guizhou Medical University, Guiyang, China

**Keywords:** Guizhou Han, Forensic features, Population genetics, InDel, X-chromosome

## Abstract

**Background:**

The insertion/deletion polymorphism (InDel), an ideal forensic genetic marker with a low spontaneous mutation rate and small amplification product fragments, is widely distributed in the genome, combining the advantages of STR and SNP genetic markers. The X-chromosome has high application value in complex paternity testing, and it is an excellent system for evaluating population admixture and studying evolutionary anthropology. However, further research is needed on the population genetics of X-chromosome InDels (X-InDels).

**Methods:**

In this article, a system composed of 38 X-InDel loci was utilized to analyse and evaluate the forensic parameters of the Guizhou Han population in order to explore its forensic application efficiency.

**Results:**

The results showed that expected heterozygosities spanned from 0.0189 to 0.5715, and the cumulative power of discrimination of the 32 X-InDels and three linkage blocks was 0.9999999954 and 0.999999999999741 for males and females, respectively. The combined mean exclusion chance of these loci for trios and duos is 0.999999 and 0.999747, respectively. Multiple methods like principal component analysis, *Fst* genetic distance, and phylogenetic reconstruction were employed for dissecting the genetic structure of the Guizhou Han population by comparing it with previously reported populations. As expected, the studied Han population displayed relatively close genetic affinities with the East Asian populations. At the same time, there were obvious genetic differentiations between the Guizhou Han population and other continental populations that were discerned, especially for the African populations.

**Conclusions:**

This study further verified the applicability of 38 X-InDels for human personal identification and kinship analyses of Han Chinese, and also showed the application potential of X-InDels in population genetics.

## Introduction

Kinship testing and individual identification are the mainstays of forensic genetic research (Butler, 2012a). Forensic researchers have also dedicated themselves to finding ideal genetic markers that are suitable for forensic genetics, including human identification, kinship analyses (Yagasaki et al., 2022), as well as non-human individual race and species identification (Carneiro, Pereira & Amorim, 2012), and bio-geographical origin inferences (Phillips, 2015). At present, short tandem repeats (STRs) typing is considered to be the most routine and authoritative method in forensic DNA identification, which can solve most practical problems and has been widely used in forensic practice (Tao et al., 2022). However, with the widespread use of STRs in forensic DNA analysis, their relatively high mutation rate and limitations in detecting degraded samples have also been exposed (Butler, 2001; Caputo et al., 2017; Sheets & Wenk, 2018). As the third-generation genetic marker, the single nucleotide polymorphism (SNP) has more and more advantages in forensic practice. Although the discriminating power (PD) of SNPs is difficult to achieve high values with STR compared, SNP has a relatively low mutation rate, and the amplification product of a single SNP locus can be obtained below 200 bp. The nature of the dimorphic marker also makes the analysis of typing results easier to perform and more automated (Oldoni, Kidd & Podini, 2019). However, the research methods for SNPs are relatively complex, with costly instruments and high usage costs. These deficiencies make the SNP locus unsuitable for promotion in regular forensic laboratories (Jian et al., 2021). To overcome the aforementioned deficiencies of STR and SNP loci, forensic scholars all over the world are searching for new genetic markers. In recent years, insertion/deletion polymorphisms (InDels), the new genetic marker, have attracted more and more attention. As a specific marker of dimorphism, InDels have a lower mutation rate and smaller fragments of PCR amplification products than STRs. Unlike SNPs, InDel is a genetic marker that is generated by the insertion or deletion of single or multiple bases in natural populations, which is widely distributed in the genome (Mills et al., 2006; Weber et al., 2002). Its advantage is that, using ordinary PCR amplification, two alleles can be distinguished by the length polymorphism of the amplification fragment (Gomes et al., 2020a). For the moment, the STR typing technology based on capillary electrophoresis, which is now routinely equipped in forensic laboratories, can be used as a technical analysis platform for InDels (Sheng et al., 2018). Moreover, relying on the size of the insertion or deletion fragment enables rapid and accurate typing, demonstrating the superiority of InDel genetic markers for forensic applications. Arguably, InDel combines the advantages of both STRs and SNPs. Therefore, InDel is considered as an ideal forensic marker and has attracted the attention of forensic researchers around the world. Studies about InDels on population genetics are now being conducted, and have been researched and used more and more for forensic genetics (He et al., 2019; LaRue et al., 2014) and biogeographic origin prediction (Bastos-Rodrigues, Pimenta & Pena, 2006).

The X-chromosome has a special inheritance pattern whereby, during female meiosis, X-chromosome markers are recombined along the entire chromosome and passed on to both female and male offspring. In males, however, the X-chromosome markers are passed exclusively to the female offspring (Butler, 2012b). Due to its unique genetic characteristics, X-chromosome genetic markers have valuable applications in paternity testing, especially in some specific cases such as “half-siblings”, “uncle and nephew”, “grandparent and grandchild” and so on (Ferragut et al., 2019; Garcia et al., 2022; Gomes et al., 2012; Medina-Acosta, 2011). Furthermore, for smaller effective population sizes, the genetic drift on the X-chromosome is faster than that of autosomal, so that genetic distances among populations on the X-chromosome are significantly larger (Schaffner, 2004). These special properties make the X-chromosome an excellent system for evaluating population admixture and studying evolutionary anthropology (Gomes et al., 2020b; Zhang et al., 2015).

The Chinese Han nationality is the largest ethnic group in the world, and its origin, development and expansion are highly complex. According to the relevant historical documents, the most widely accepted view today is that the origin of the Han nationality can be traced back to the Huaxia ethnic group in the Central Plains of China during the Shang and Zhou dynasties (21st-8th century BC) (Cioffi-Revilla & Lai, 1995). The Han Dynasty ruled for 405 years, during which time the Huaxia ethnic group developed into a tribe known as the Han people (Ruofu, 1993). They first lived in central China and then gradually merged with the eastern and southern parts of China. Historically, there have been regional genetic differences among Han Chinese as a result of ethnic integration (Yang et al., 2017). So far, genome-wide studies have also shown that the Chinese Han population can be divided into two distinct populations, the Southern and Northern Han (Qu et al., 2012). Currently, according to data from China’s seventh national population census, Han Chinese account for 91.11% of China’s total population. In the hinterland of southwest China’s interior sits the multiethnic province of Guizhou. It serves as a transportation hub for southwest China and is crucial to the Yangtze River Economic Belt (Zelong et al., 2020). The total population of Guizhou Province is 38.52 million, of which the Han Chinese population accounts for 62.2% of the total population. The Guizhou Han in this study belongs to the Southern Han Chinese. For the first time, 38 X-InDel loci of 264 Guizhou Han individuals were genotyped and forensic parameters were calculated, providing basic population data for parentage and individual identification. In addition, the 38 X-InDel loci were used to explore the genetic affinities between the Guizhou Han population and 27 reference populations, including the 1,000 Genomes Project (Durbin et al., 2010; Zhang et al., 2021).

## Materials and Methods

### Sample collection

Fingertip blood samples were collected from 264 unrelated Guizhou Han people (128 females and 136 males) in accordance with the principle of informed consent. After explaining the objectives and procedures of our study, all participants provided their written informed consent. Ethical permission was warranted by the Ethics Committee of Guizhou Medical University (Approval Number: No. 2021-218), and followed the recommendations provided by the revised Helsinki Declaration of 2013.

**Figure 1:**
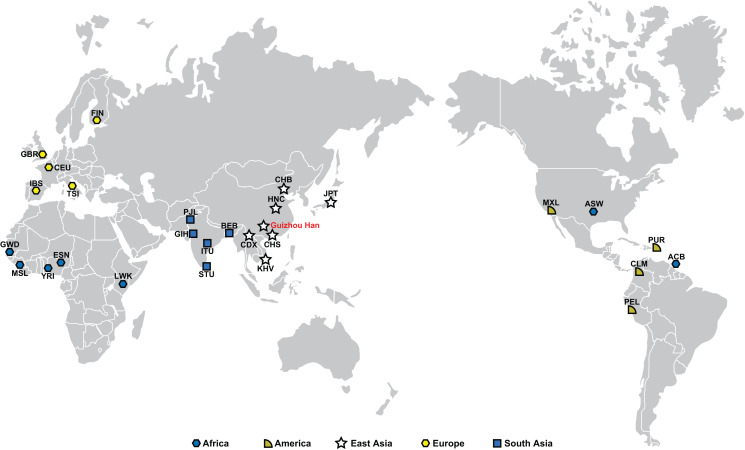
Guizhou Han population and other 27 worldwide reference populations’ geographic locations. Populations are represented by dots in their respective positions, and five continents are represented by five different colors. The population names are abbreviated as follows: ASW, African Ancestry in Southwest US; ACB, African Caribbean in Barbados; GWD, Gambian in Western Division, The Gambia—Mandinka; MSL, Mende in Sierra Leone; YRI, Yoruba in Ibadan, Nigeria; ESN, Esan in Nigeria; LWK, Luhya in Webuye, Kenya; FIN, Finnish in Finland; GBR, British in England and Scotland; CEU, Utah residents (CEPH) with Northern and Western European ancestry; IBS, Iberian populations in Spain; TSI, Toscani in Italy; PJL, Punjabi in Lahore, Pakistan; GIH, Gujarati Indians in Houston, TX; ITU, Indian Telugu in the UK; STU, Sri Lankan Tamil in the UK; BEB, Bengali in Bangladesh; CDX, Chinese Dai in Xishuangbanna, China; CHS, Han Chinese South; HNC, Han Chinese, Henan, China; CHB, Han Chinese in Beijing, China; JPT, Japanese in Tokyo, Japan; KHV, Kinh in Ho Chi Minh City, Vietnam; MXL, Mexican Ancestry in Los Angeles, California; PUR, Puerto Rican in Puerto Rico; CLM, Colombian in Medellin, Colombia; PEL, Peruvian in Lima, Peru.

At the same time, we also collected the raw data and allele frequencies of 38 X-InDels from 26 populations in the 1,000 Genomes Project (Durbin et al., 2010) and the previously published Han Chinese in Henan province (HNC) (Zhang et al., 2021), as reference populations covering five continents worldwide. The geographical distribution of the study and reference populations was visualised using the ggplot2 package in the R 3.3.0 software (https://www.r-project.org/), as shown in Fig. 1.

### PCR amplification and genotyping

Without DNA extraction, all blood samples were directly amplified. The Thermo 96-Well PCR System (Thermo Fisher Scientific, Waltham, MA, USA) was used to genotype a total of 264 unrelated samples. A total of 38 X-InDel markers were included in the analysis panel. The specific procedures for PCR and the general information of different markers were described in a previous publication (Chen et al., 2021). Isolation of amplification products was performed *via* the ABI 3500xL Genetic Analyzer (Applied Biosystems, Foster City, CA, USA). The GeneMapper v 4.0 was used to perform electropherogram analysis and allele assignments.

### Statistical analysis

The 38 X-InDel loci were examined for the Hardy-Weinberg Equilibrium (HWE) using the Genepop 4.7 software package (Rousset, 2008). In addition, the linkage disequilibrium (LD) was tested and visualised by the SNP analyzer software (Kling, 2017), and the Bonferroni procedure was also used to correct the *p* values. HWE was assessed in females, and LD was tested by combining the Chi-square test and *p*-values from male haplotype counts (Caputo et al., 2017). We estimated allele frequencies of 38 X-InDel loci and forensic-related parameters of 32 X-InDel loci by StatsX v2.0 software (Lang, Guo & Niu, 2019). In addition, haplotype frequencies and forensic parameters of three linkage blocks were also estimated by the StatsX software. To compare the allele frequencies between males and females, Arlequin v3.5.2.2 software (Excoffier, Laval & Schneider, 2005) performed Fisher’s exact test.

The genetic distances (*F-statistics, Fst*) were calculated by the Genepop 4.7 software package (Rousset, 2008) based on genetic profiles of 38 X-InDel loci and visualised *via* the R 3.3.0 software (https://www.r-project.org/). MVSP 3.22 software was used to perform allele frequency-based principal component analysis (PCA) to obtain a better understanding of the demographic relationships of the different populations. With the data from the *Fst* genetic distance matrix, MEGA software v7.0 (Kumar, Stecher & Tamura, 2016) was utilised to construct the neighbour-joining (NJ) tree.

## Results

### Allele frequencies of 38 X-InDels in the Guizhou Han population

**Figure 2:**
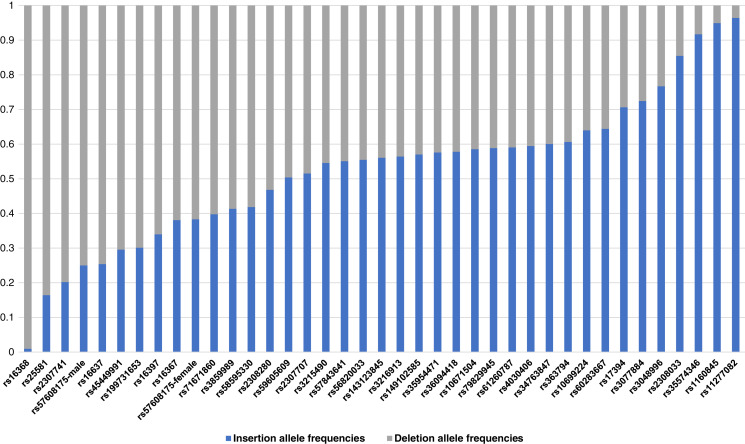
A histogram of allele frequencies at the 38 X-InDel loci of the Guizhou Han population. The histogram was drawn according to the insertion/deletion allele frequency of each locus. The blue bar represents the insertion allele frequency, and the grey bar represents the deletion allele frequency.

The raw genotype of the 38 X-InDel loci for 264 individuals of the Guizhou Han Chinese are shown in Table S1. The insertion/deletion allele frequencies were calculated, as shown in Fig. 2 and Table S2. Based on Fisher’s exact test, the genetic differentiations of 38 X-InDel loci between males and females were analysed for allele frequencies. With the exception of the rs57608175 locus (*p* = 0.00732), there were no discernible differences between males and females for these X-InDels (*p* > 0.05). The allele frequencies were calculated separately for males and females of the rs57608175 locus based on the results of the Fisher’s exact test, and the allele frequencies of the remaining 37 X-InDel loci were calculated by combining males and females. We found that the minor allele frequencies of most loci were larger than 0.2. In addition, a total of 136 unique haplotypes are observed in all males for these 38 X-InDels and no shared haplotypes are discerned among these individuals. Therefore, the haplotype diversity of 38 X-InDels in males is 1.

### HWE and LD analyses of 38 X-InDels in the Guizhou Han population

**Figure 3:**
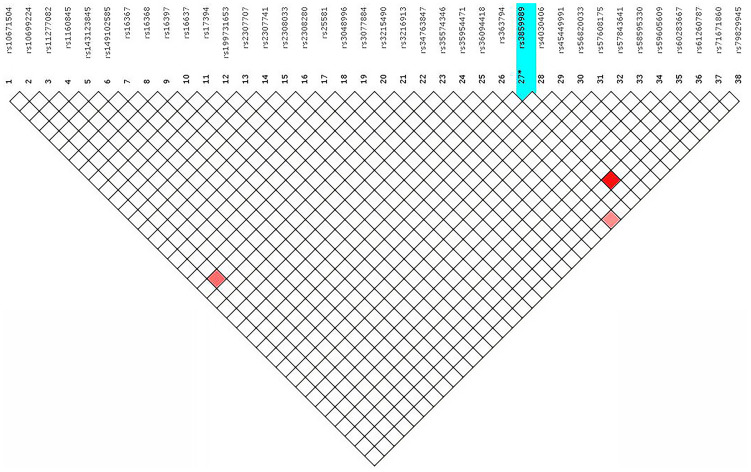
Linkage disequilibrium among the 38 X-InDel loci in the Guizhou Han population. The colour depth of the red represents the degree of linkage disequilibrium.

The 38 X-InDels typing data of 264 Guizhou Han samples were analysed with the help of HWE and LD. It was found that the two loci, rs56820033 and rs45449991, did not conform to HWE (*p* < 0.05). Even so, after the Bonferroni correction (*p* > 0.05/38), there was only one p-value for the rs56820033 locus that was not within the allowable range (Table S3). As shown in Fig. 3, LD tests were performed to illustrate the presence of LD in these 38 X-InDel loci. Combined with the Chi-square test significance, under the condition of r^2^ ≥ 0.8 and *p* > 0.05/703 (after Bonferroni correction), LD test results showed that rs3859989 and rs61260787, rs36094418 and rs79829945, rs3216913 and rs10699224 formed three linkage blocks, respectively. In accordance with the recommendations of the DNA commission of the ISFG on X genetic markers, pairs of loci must be analysed together (Tillmar et al., 2017). The two loci in each block were combined to calculate the haplotype frequencies and used for subsequent forensic parameters calculation.

### Forensic parameters of 38 X-InDels in the Guizhou Han population

The forensic application capability of the 38 X-InDel systems in the Guizhou Han population was evaluated, and the forensic parameters were calculated by the StatsX v2.0 software, and the results are shown in the Table S4. Based on the results, it can be seen that the He of 32 X-InDels in the Guizhou Han Chinese is between 0.0189 and 0.5009; MEC_Desmarais is in the range of 0.0186 to 0.3750; and moreover, MEC_Desmarais_duo ranges from 0.0094 to 0.2500. Among them, the loci rs16368, rs11277082, rs1160845, rs35574346, rs2308033, and rs25581 have generally low forensic application values. The combined power of discrimination (CPD) of the 32 X-InDel loci and three linkage blocks was 0.9999999954 and 0.999999999999741 for males and females in the Guizhou Han population, respectively. The combined mean exclusion chance (MEC) of these loci is 0.999999 in trios and 0.999747 in duos. Using 0.9999 as the standard, the outcomes demonstrated that these loci could satisfy the requirements of forensic personal identification and paternity testing in the Guizhou Han Chinese.

### Allele frequency distributions of 38 X-InDels in 28 populations

**Figure 4:**
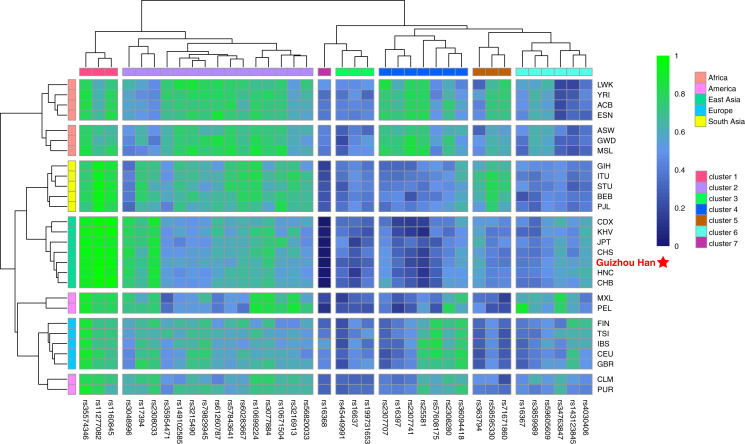
Heatmap on the basis of the insertion allele frequency distributions for the Guizhou Han population and other 27 reference populations worldwide. Blue represents low insertion allele frequency and green represents high insertion allele frequency. According to their geographical location, 28 populations are divided into five continental clusters. In addition, 38 X-InDel loci were divided into seven clusters.

The raw data of the 38 X-InDel loci from the Guizhou Han population and 27 reference populations worldwide were merged and visualised as a heatmap based on the insertion allele frequencies, as shown in Fig. 4. It is clearly displayed that the distribution of allele frequencies of the 38 X-InDel loci in various continental populations. Low insertion allele frequencies were represented by blue, whereas high insertion allele frequencies were represented by green. The heatmap results showed that all populations are clustered into five groups according to different continental origins, except for CLM and PUR, which are clustered with the European group on the same branch; moreover, the Guizhou Han population in our study formed a branch with East Asian populations. The 38 X-InDel loci were classified into seven clusters by cluster analysis. The cluster one includes rs35574346, rs11277082, and rs1160845 loci, which have an extremely high insertion allele frequency (>0.9) in the East Asian populations. Conversely, the only rs16368 locus in cluster seven has low insertion allele frequencies in all populations, especially in the East Asian population (0–0.035). Cluster two contains 15 loci, and the average insertion allele frequencies of these 15 X-InDel loci in 28 populations range from 0.55 to 0.72, which are all relatively high polymorphic in all populations. The insertion allele frequencies of the remaining clusters vary considerably among different continental groups. Genetic markers with large allele frequency differences among populations from different geographical regions or origins could be considered to be ideal ancestry informative markers (AIMs). Based on the results observed in Fig. 4, it was found that loci rs16368 and rs25581 had particularly low insertion allele frequencies in the East Asian populations. In contrast, loci rs11277082 and rs1160845 had extremely high insertion allele frequencies in the East Asian and South Asian populations. For locus rs2307741, there was a considerably high insertion allele frequency in the African populations. At the same time, locus rs16367 displayed a very high insertion allele frequency in the PEL population from the America, and locus rs34763847 had a higher allele frequency in both the PEL and MXL populations from the America than those in other continental populations.

### Exploration of genetic structure and genetic affinity of Guizhou Han and other reference populations based on 38 X-InDels

**Figure 5:**
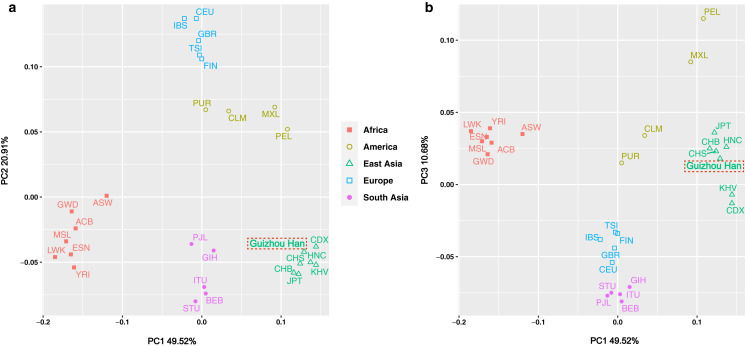
PCA plot among the 28 populations based on the allele frequencies of 38 X-InDel loci. Populations are classified according to their geographical location and identified using different colours and shapes. Among them, the green triangle represents the East Asian clusters, and the Guizhou Han population in this study is marked with red dotted frames.

The insertion allele frequencies-based PCA was carried out in order to more intuitively reveal the genetic structure of the Guizhou Han population and highlight the genetic affinities between the studied population and the 27 worldwide reference populations. Meanwhile, the scatter diagram was drawn based on the contribution rates of the first three principal components in the PCA results, as shown in Figs. 5A and 5B. Of these, the contribution rates of PC1, PC2 and PC3 were 49.52%, 20.91% and 10.68%, respectively. These three principal components concentrated on explaining about 81.11% of the genetic structural variances among populations. The 28 populations were classified at the continental level into five major groups: America, Africa, East Asia, South Asia, and Europe, and labelled with five different colours and graphics. From the overall view of Fig. 5A, the populations from each continent were clustered significantly, in the shape of a “cross”. It is worth noting, however, that the aggregation degree of the American populations was lower than that of the groups from the other four continents, showing closer genetic affinities with the European populations.

Although in general, the East Asian populations including Guizhou Han, CDX, CHB, CHS, JPT, KHV, and HNC were tightly clustered in the lower right quadrant. However, according to the substructure exhibited by PC1, Guizhou Han was more closely clustered with CHS and HNC, and has a closer genetic affinity. On the other hand, the PC3 could differentiate South Asian and European populations from other populations (Fig. 5B).

**Figure 6:**
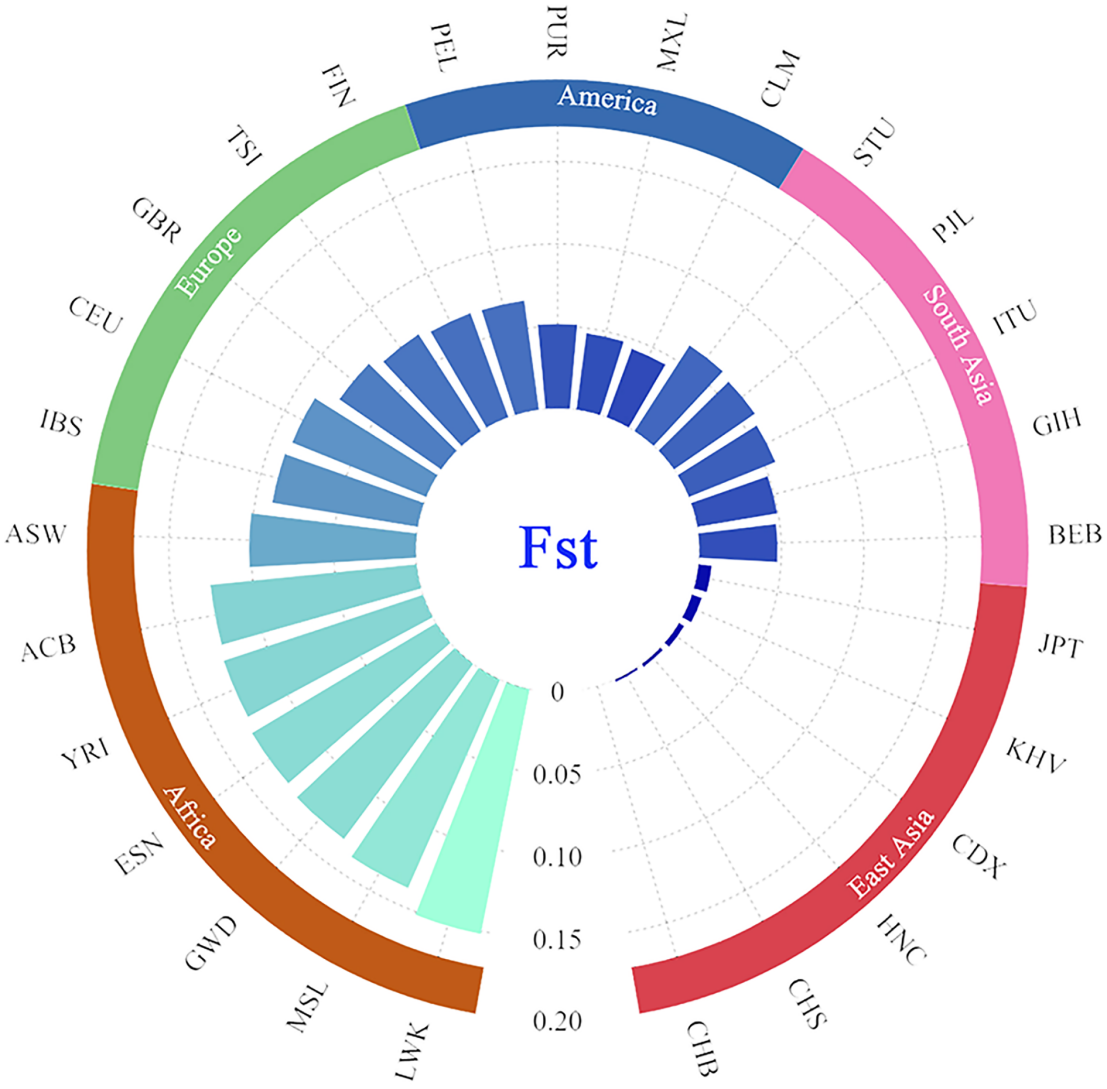
Grouping histogram on the basis of *Fst* genetic distances between the Guizhou Han population and the other 27 reference populations. The depth of blue and the length of the histogram represent the *Fst* genetic distance. The reference populations are grouped according to their geographical location.

In addition, the paired *Fst* genetic distances were calculated among the Guizhou Han and other compared populations. The results were presented in the form of a grouping histogram (Fig. 6).

Combined with Fig. 6, according to the geographical location, it is clear that the genetic affinities between the Guizhou Han population and the populations in the East Asian region were close, especially for the Beijing Han Chinese (CHB, *Fst* = 0.000), South Chinese (CHS, *Fst* = 0.0018) and Henan Han Chinese (HNC, *Fst* = 0.0024). On the contrary, the genetic affinities between the Guizhou Han population and the African populations were the furthest (with the largest genetic distance), especially with the Luhya in Webuye, Kenya (LWK, *Fst* = 0.1519).

**Figure 7:**
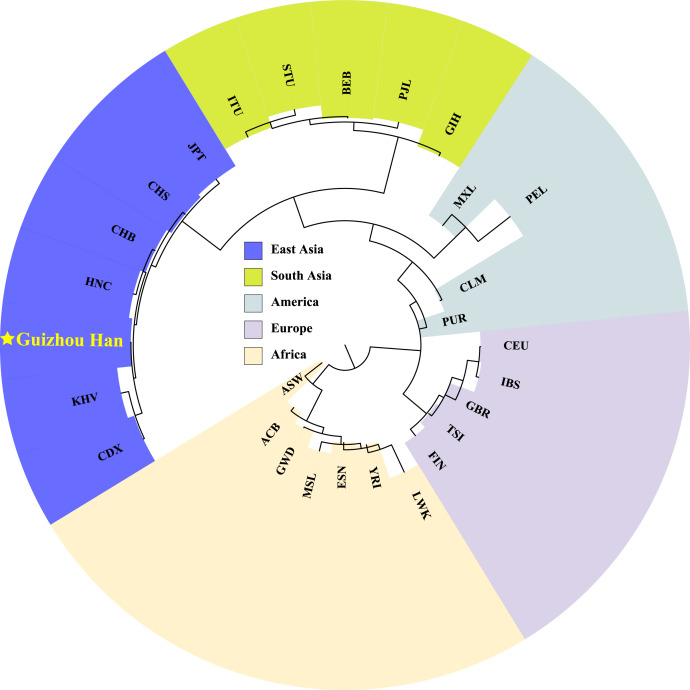
Neighbor-joining phylogenetic tree constructed on the basis of the *Fst* genetic matrix among the 28 populations. The NJ tree, constructed according to *Fst* genetic distance, uses different colour blocks to represent different geographical locations and a red five-pointed star to highlight the Guizhou Han population in this study.

In order to explore phylogenetic relationships among the Guizhou Han population and other reference populations, we constructed a neighbor-joining (NJ) phylogenetic tree based on their pairwise *Fst* genetic distances, as shown in Fig. 7. The results of the NJ tree construction support the population genetic relationships where the Guizhou Han was classified from the same branch as CHB, and then clustered with CHS and HNC in the same branch.

## Discussion

The analysis of allele frequencies of the 38 X-InDel loci showed they are evenly distributed in the Guizhou Han population. However, the allele frequency distribution at locus rs57608175 showed a significant difference between males and females. The same situation has not been found in other studies (Chen et al., 2021; Zhang et al., 2021), which may be due to an insufficient sample size. Perhaps in a follow-up study, the sample size could be increased for further in-depth exploration. The results of LD tests indicated that the 32 X-InDels (except loci rs3859989, rs61260787, rs36094418, rs79829945, rs3216913, and rs10699224) in the Guizhou Han population do not show significant association. Previously published studies of Henan Han Chinese also showed the presence of LD in rs3859989 and rs61260787, rs36094418 and rs79829945, rs3216913 and rs10699224 (Zhang et al., 2021). Furthermore, the results of the HWE test showed that locus rs56820033 deviated from the HWE, which was not observed in an another study (Zhang et al., 2021) with the same X genetic marker panel. The explanations for the LD test results may be grounded on patterns and degrees of genetic admixture, population genetic structure, or locus selection. In any case, the sample size needs to be increased (Martinez et al., 2019). In addition, the HWE deviation of locus rs56820033 is disturbing and genotyping errors due to polymorphism in the primer binding sequence must be further investigated.

According to the statistical calculation results of forensic parameters, it is revealed that the combined PDs and MECs of the Guizhou Han population calculated by the 38 X-InDels panel are higher than those in alternative X-InDels studies (Chen et al., 2021; Edelmann et al., 2016; Freitas et al., 2010; Pereira et al., 2012; Zhang et al., 2021), although lower than those of an X-STRs panel (Liu et al., 2018). Even so, due to the lower mutation rate of InDels, it can provide valuable information in complex, deep genealogies testing. Furthermore, we observed that the loci rs16368, rs11277082, rs1160845, rs35574346, rs2308033, and rs25581 have relatively low genetic diversities in Guizhou Han Chinese, similar findings having been reported in another East Asian population (Zhang et al., 2021), indicating that these loci may possess relatively low genetic diversities in Han populations. To sum up, we propose that the panel of 38 X-InDel loci can be used as an effective auxiliary tool for individual identification and paternity testing in China, as well as an effective supplementary detection system in complex kinship testing that cannot be resolved by autosomal STRs.

The distribution of allele frequencies in 28 populations showed that the loci rs16368, rs25581, rs11277082, rs1160845, rs2307741, rs16367, and rs34763847 have great potential as ideal AIMs. Particularly in African, American, and East Asian populations, it appears that the majority of the 38 X-InDel loci can manifest relatively high genetic divergences, which is of great utility for forensic ancestry origin analyses.

The results of PCA analysis and *Fst* genetic distance showed that the genetic distances between the Guizhou Han and other populations in the same East Asian region are relatively small, and studies on the autosomal or Y-chromosomes STRs of Guizhou Han Chinese also showed similar clustering patterns (Chen et al., 2018; Yang et al., 2017). It has been shown that genetic affinities are closely related to geography and language (Abdulla et al., 2009; Su et al., 1999; Watanabe et al., 2019), which is also consistent with our results.

## Conclusions

In our study, based on the newly built 38 X-InDels system, the X-InDels typing data of 264 individuals from the Guizhou Han population was analyzed, and the forensic application effectiveness of the system was explored and verified. The statistical results of the forensic parameters indicate that the system can be used as an excellent auxiliary tool for the forensic paternity testing and individual identification of Han Chinese, which also support its potential use in population genetics. Population genetic analyses of Guizhou Han and other reference populations showed Guizhou Han population had relatively close genetic affinities with Han populations from East Asia. In addition, some loci out of these 38 X-InDels could be viewed as candidate AIMs for distinguishing different continental populations. In summary, the data in this study have laid the groundwork of demographic data for the use of the 38 X-InDels in individual identification, kinship testing and biogeographic ancestry analysis.

## Supplemental Information

10.7717/peerj.14964/supp-1 Supplemental Information 1 Raw data of 38 X-Indels in the Han Chinese population of Guizhou.

10.7717/peerj.14964/supp-2 Supplemental Information 2 Allele frequencies of 38 X-Indels loci in Guizhou Han population.

10.7717/peerj.14964/supp-3 Supplemental Information 3 Analysis of Hardy-Weinberg test of 38 X-InDel loci.

10.7717/peerj.14964/supp-4 Supplemental Information 4 Analysis of forensic statistic parameters of 32 X-InDels and three linkage blocks in the Guizhou Han population.

## Additional Information and Declarations

### Data Availability

The following information was supplied regarding data availability: The raw measurements are available in the Supplemental Files.
